# Hearing Health at School: analysis of knowledge, behaviors and attitudes of Southern-Brazilian children and adolescents on noise

**DOI:** 10.1590/2317-1782/20242023181en

**Published:** 2024-08-02

**Authors:** Lys Maria Allestein Gondim, Débora Lüders, Milena Kovalski Oliveira, Cristiano Miranda de Araújo, Adriana Bender Moreira de Lacerda

**Affiliations:** 1 Universidade Tuiuti do Paraná – UTP – Curitiba (PR), Brasil.; 2 Université de Montréal, Montréal, Quebéc, Canada.

**Keywords:** Noise, Health Risk Behaviors, Tinnitus, Noise-induced Hearing Loss, School Health Services

## Abstract

**Purpose:**

to analyze the knowledge, behaviors, and attitudes of students on noise.

**Methods:**

We used an observational method study, in 32 Schools from Itajaí/Brazil, with a convenience sample, comprising 1,835 students, 45.7% females and 54.3% males, mean age of 11.53 ± 0.8 years, was conducted. The Portuguese version of Dangerous Decibels® questionnaire was applied. For the data analysis, descriptive and inferential statistics were used, significance level of 5%.

**Results:**

A large part of the sample part of the sample had harmful hearing habits; 62.6% reported knowledge on the subject, but only 25.4% answered the questions on the theme correctly; 58.9% reported that they did not know how to protect hearing if necessary. Regarding sex and age: males have more hazardous hearing habits (p < 0.001) and tinnitus complaint (p<0.001) and females have more knowledge (p < 0.001) and the intent to wear hearing protection (p<0.001), greater intention to wear hearing protection among younger students (10 and 11 years old) (p < 0.001), and the older ones (12 to 16 years old) get more exposed to noise (p < 0.001), and there are more tinnitus complaints (p < 0.05) among them.

**Conclusion:**

A large part of the students in the study has hazardous noisy habits and scarce knowledge on the theme, with males and older subjects showing the worst attitudes and behaviors in face of the potential hearing risk caused by exposure to high noise levels.

## INTRODUCTION

The World Health Organization (WHO) reported that 60% of child hearing loss is preventable, and about 1.1 billion young people between 12 and 35 years old are at risk of noise-induced hearing loss (NIHL) due to entertainment environments, attendance to concerts, the use of earphones, games etc^(1)^.

In modern society, the use of cell phones, tablets, and the like, in addition to powerful stereo equipment and earphones has astonishingly increased. The attendance to venues with loud music has been increasingly common. Options of leisure for the youth, with noise exposure, have become a habit and considered a socially accepted health hazard. Children, adolescents, and young adults are the most exposed to environmental noise and a trend for increasing noise-induced hearing loss (NIHL) has been observed among that group in several studies^(2-6)^.

The lack of visibility and expansion of knowledge, little value, and knowledge on noise effects in entertainment activities are still factors contributing to the increasing risk and development of NIHL. As it is a preventable loss, its approach should start by the awareness of the younger population^(1,7-10)^.

To consider further directions in the elaboration of educational interventions and hearing health promotion, there is the initial need of searching for information on knowledge, behaviors and hearing habits of children and adolescents in face of noise. Thus, the goal of this study was to analyze students’ behaviors, habits, knowledge, and attitudes on noise.

## METHODS

A monocentric, observational, transversal study was conducted with 6th graders comprising all 32 Municipal Public Schools from Itajaí, Santa Catarina State, Southern Brazil, subdivided into 8 Educational Pools. In 2021 the city's population was 264,054 and the average monthly wage of workers was 3.0 minimum wages. The study was approved by the Ethics Board of Research, the Tuiuti do Paraná University, opinion number 2,551,067. All participants signed an informed consent form.

### Sampling

The calculations for the sampling size were based on all 2,160 students enrolled in the 6th grade, considering an infinite population, the calculated minimum sample comprised 1,068 students, 95% confidence level and sampling error of 3% (α=0.03).

The sampling loss was 15% (325 students), that is, 12% (39 students) did not sign the Free Informed Assent Form for Minors and/or the Free Informed Consent Form for their legal guardians, and the other 88% (286 students) were not present in all the steps of the research (therefore, they did not respond the questionnaires). The final sample comprised 1,835 students, with 838 (45.7%) females and 997 (54.3%) males, mean age of 11.53 ± 0.8 years.

To assess the age-related data, the study group was divided into two categories^(11)^: children at ages between 10 and 11 years and adolescents at ages between 12 and 18 years. This distribution was based on the Statute of the Child and Adolescent (ECA). According to this statute, children are those up to 12 years of age. Those aged between 12 and 18 are adolescents.

### Instruments

To analyze students' behaviors, habits, knowledge and attitudes towards noise, was used a questionnaire based on the validated questionnaire ^(12)^ from the Dangerous Decibels® Program (DD) (Chart 1). The original questionnaire ^(12)^ was translated and adapted into Portuguese but was not validated ^(13).^ In addition, other questions about classroom noise have been added in the Brazilian study ^(13).^ In the current study, these additional questions were not included.

**Chart 1:** Detailed questionnaire used in the study, according to its dimensions (habits, symptoms, behavior, attitudes, knowledge, opinion and general information)

**ISSUES**
1. Risk habits/exposure to loud sound & noise (use of headphones, frequency of music shows, playing in a band, riding in a car equipped with powerful sound, exposing yourself to fireworks, going to a noisy car show, going to a car or motorcycle race etc.) (H)
2. Complaint of earache or tinnitus when exposed to loud sound/noise(S)
3. Habit of wearing hearing protectors when exposed to loud sound/noise (B)
4. Which sounds are loud enough to impair the hearing (headphones, music shows, fireworks etc.) (H)
5. Good ways to protect hearing when exposed to loud sound/noise (move away from noise, make use of hearing protectors, lower volume etc.) (H)
6. I am aware of sounds that can cause hearing loss (O)
7. I am aware of how to protect my hearing (O)
8. Hearing an extremely loud sound, even if only once, can lead to loss of part of the hearing (K)
9. Very loud sounds can damage the small hair cells of the inner ear (K)
10. Hearing loss is a problem only for the elderly (K)
11. People with hearing loss often have difficulties in (hearing alarms, doorbell or phone ringing; getting a job, understanding what is said in the classroom or in a group etc.) (O)
12. Having a hearing loss is not a problem (A)
13. People who listen to loud music too long seem to have no hearing loss, so I don´t have to worry about having a hearing loss either (A)
14. If I go to a show with loud music, I`ll wear a hearing protector (B)
15. Tinnitus complaint (S)
16. I have participated in some campaign/educational action about hearing at school (G)
17. Sex (IG)
18. Age (IG)

**Caption:** (H)=Habits; (S)= Symptoms; (B)= Behavior; (A)=Attitudes; (K)= Knowledge; (O)=Opinion; (IG)-General Information

The questionnaire used consisted of 18 structured questions, printed on a single page, including general data (age and gender), questions involving knowledge about sound, noise, hearing, and hearing protection strategies, as well as habits and symptoms, attitudes, and behaviors in relation to noise (Chart 2).

**Chart 2:** Questionnaire translated and adapted into Brazilian Portuguese by Knobel and Lima (2014)^(13)^ used in this study

**1. Durante o último ano eu:**		
*Marque todos que se aplicam a você*		
◻ Usei Fones de ouvido ou MP3 player		
◻ Usei um cortador de grama		
◻ Andei em uma moto		
◻ Fui a uma apresentação de carros barulhentos		
◻ Andei em um carro equipado com som potente no porta-malas		
◻ Toquei em uma banda		
◻ Fui a uma apresentação de carros barulhentos		
◻ Andei em um carro equipado com som no porta-malas		
◻ Toquei em uma banda		
◻ Fui a uma corrida de carros ou motocicletas		
◻ Fui a um show de música		
◻ Usei fogos de artifício		
**2. Durante o último ano, eu estive perto de sons altos que doeram meus ouvidos ou causaram um zumbido**		
**□** Sim	**□** Não	**□** Não tenho certeza
**3. Eu uso o protetor auditivo quando estou próximo a som alto**		
**□** Sim	**□** Não	**□** Não tenho certeza
4. **Quais dos seguintes sons são altos o suficiente para prejudicar sua audição?**		
◻ Fones e MP3 players		
◻ Máquina de lavar pratos		
◻ Fogos de artifícios		
◻ Máquina de lavar roupas		
◻ Shows		
*5.* **Quais dessas maneiras são boas para proteger sua audição quando você está próximo de sons altos?**		
*Marque todos que se aplicam a você*		
◻ Afastar-se do som alto		
◻ Colocar algodão ou lenço de papel no ouvido		
◻ Diminuir o volume		
◻ Usar protetores auriculares		
◻ Escutar sons altos por longos períodos para acostumar-se		
◻ Sempre que possível, passar menos tempo próximo a sons altos		
6. **Eu tenho conhecimento quanto aos sons que podem causar perda auditiva**		
**□** Verdadeiro	**□** Falso	**□** Não tenho certeza
7. **Eu tenho conhecimento de como proteger minha audição quando estou próximo a som alto**		
**□** Verdadeiro	**□** Falso	**□** Não tenho certeza
**8. Ouvir um som extremamente alto, mesmo que apenas uma vez, pode levar a uma perda de parte de sua audição**		
**□** Verdadeiro	**□** Falso	**□** Não tenho certeza
9. **Som muito alto pode danificar as pequenas células ciliadas do ouvido interno.**		
**□** Verdadeiro	**□** Falso	**□** Não tenho certeza
10. **Perda auditiva é um problema somente de idosos**		
**□** Verdadeiro	**□** Falso	**□** Não tenho certeza
11. **Pessoas com perda auditiva geralmente tem dificuldades com o seguinte:**		
◻ Ouvir alarmes, campainha da porta ou telefone tocando		
◻ Entender sinais de sinalização de rodovias		
◻ Entender o que é dito em um grupo		
◻ Conseguir um trabalho		
◻ Entender o que é dito em filmes, peças de teatro e TV		
◻ Entender o que é dito na sala de aula		
12. **Ter uma perda auditiva não é um problema**		
**□** Concordo	**□** Discordo	**□** Não tenho certeza
13. **Pessoas que escutam música alta todo o tempo parecem não ter perda auditiva então eu não tenho que me preocupar**		
**□** Concordo	**□** Discordo	**□** Não tenho certeza
**14. Se eu for a um show com música alta, eu irei usar um protetor auditivo**		
**□** Sim	**□** Não	**□** Não tenho certeza
**15. Você sente algum zumbido ou outro tipo de barulho em seu ouvido ou na cabeça?**		
**□** Sempre	**□** Às vezes	**□** Nunca
**16. Você já participou de alguma campanha sobre audição na escola?**		
**□** Sim	**□** Não	**□** Não tenho certeza
**17. Você é :**		
**□** Menino	**□** Menina	**□** Prefiro não responder
**18. Qual sua idade? ____**		

The questionnaire was applied by the teachers at the schools, always monitored by the researcher, in their respective classes, in the usual school shifts of the students. The teachers went under previous training to the application, conducted by the lead researcher, with detailed explanation of each question. The questionnaire was handed out to each student and read aloud to all the students, pausing for their responses to each question. In case of doubts, the teacher and/or the researcher would help the students. Average time to answer the questionnaires was 15 minutes.

### Statistical analyses

For the analysis of the individual results, descriptive statistical methods were used (with absolute and relative frequency tables in percentage - %). For the comparisons of the questions regarding sex and age range, Chi-square Test were used, significance level of 0.05 (5%), considering only the affirmative responses (yes, always, true, agree) and negative ones (no, never, false, disagree) for the test application. *Statistica* 13.3 software was used for the analyses.

## RESULTS

In the current study 96.9% of the participants reported that they had never participated in any educational campaign or hearing health promotion program in their schools (whether the current ones or the former ones when the research was carried out).

**Figure 1:**
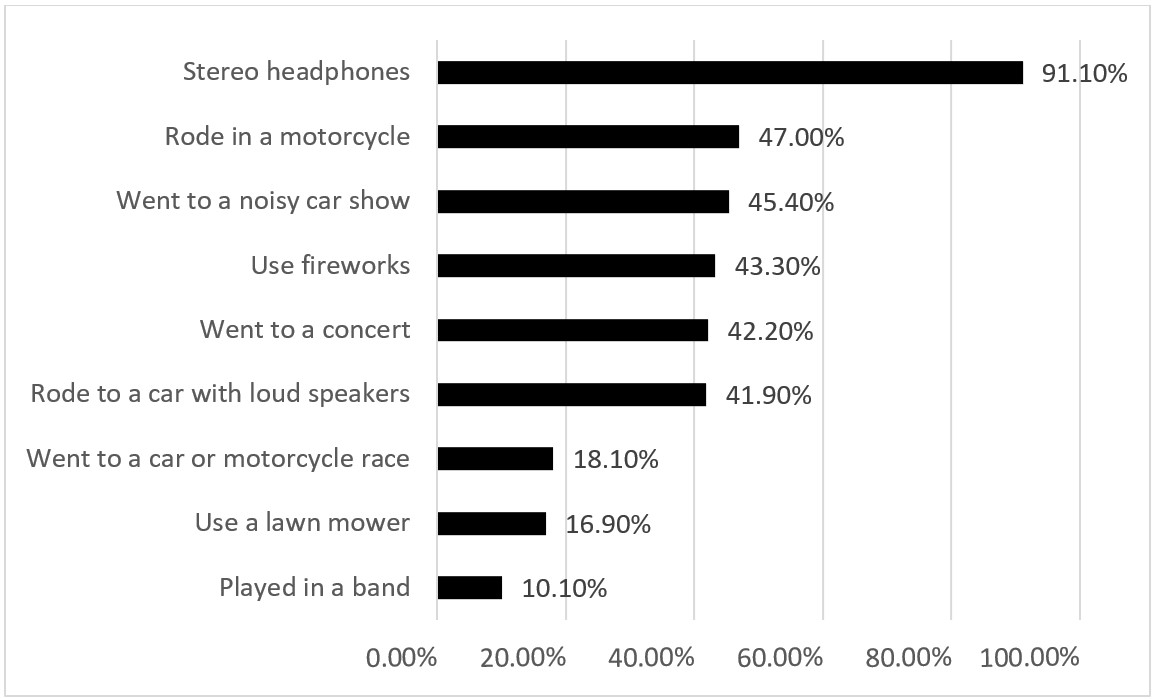
Risk hearing habits – percentage of participants exposed to common noisy sound sources

Regarding the risk habits, the descriptive analyses verified that 91% of students have the habit of using stereo headphones, but in other habits, the occurrence is less than 50%, such as rode in a motorcycle (47%), went to a noisy car show (45.40%), use fireworks (43.30%); went to a concert (42.20%), rode in a car with loud speakers (41.90%), went to a car or motorcycle race (18.10%), use a lawn mower (16.90%), playing in a band (10.10%) (Figure 1).

Concerning otological or hearing symptoms and signs, 47.6% of the adolescents reported otalgia or tinnitus when exposed to loud noise levels, and 72.2% of them had tinnitus complaint.

Regarding habits, knowledge, attitudes, behavior, and tinnitus complaint, part of the results is shown in Table 1.

**Table 1:** Percentage data and statistical differences (by sex / female and lower age group) of the answers, based on the results of the questionnaires, according to the scales of habits, knowledge, attitudes and behavior and tinnitus complaint

**ISSUES**	**%**	***p* (sex)**	***p* (age)**
**HABITS**			
1. Types of sounds loud enough to impair hearing:			
- headphones	47.3	*0.0000	0.3733
- shows	58.0	1.0000	*0.0145
2. Good ways to protect hearing when close to loud sound – noise:			
- move away from loud sound	77.3	0.3341	0.1179
- decrease the volume	80.2	0.1196	0.2008
- wear hearing protectors	34.1	*0.0009	*0.0022
**KNOWLEDGE**			
1. Hearing an extremely loud sound, even if only once, can lead to loss of part of the hearing	44.3	*0.0000	*0.0034
2. Too loud sound can damage the small hair cells of the inner ear	65.1	*0.0006	0.3263
3. Hearing loss is a problem only of the elderly	14.3	*0.0035	0.1020
**ATTITUDES**			
1. Having a hearing loss is not a problem	81.5	0.9308	0.0578
2. People who listen to loud music too long seem to have no hearing loss, so I don't have to worry	56.2	*0.0000	*0.0000
**BEHAVIOUR**			
1. I wear hearing protectors when I'm near loud sound – noise	10.3	0.7914	0.0565
2. If I go to an event with loud music, like a music show, I will wear hearing protectors	18.1	^*^0.0000	*0.0001
**TINNITUS COMPLAINT**	72.7	*0.0056	*0.0256

* Difference test of proportions at significance level of 0.05 (5%)

**Caption:** %: percentage (general responses); *p* (sex): results of '*p*' - difference between female and male and *p* (age): results of '*p'* - difference between the age groups lower (10 and 11 years of age) and higher (12 to 16 years of age)

Concerning knowledge on the perceptions of the difficulties that hearing-impaired people may face, 83.1% stated that they would usually have difficulties in hearing alarms, doorbells or phone ringing; 65% thought that they would have difficulties in understanding what was said in a group (in a conversation); 43.1% in getting a job; 66.9% in understanding what would be said in a movie, play or on the television; 75.8% in understanding what would be said in a classroom, and 17.1% also associated hearing loss with difficulties in understanding traffic signs along the highways.

As for habits and knowledge on noise, 62.6% admitted that they did not have any knowledge on noise-related hearing loss, and 58.9% reported that they did not know how to protect their hearing if necessary, calling attention to the use of hearing protection devices as a protection strategy, reported by only 34.1% of the studied sample. Moreover, among the students who reported knowledge on what kind of noise could cause hearing loss (37.4%), only 25.4% answered the questions on the theme correctly. In addition, as for knowledge on how to protect hearing, the students who claimed that they had such knowledge (41.1%), only 23.1% responded the specific questions on the theme correctly.

Concerning attitudes and behaviors, 81.5% of the participants in the study consider that developing hearing loss is not a problem, and only 10.3% wear hearing protection devices when exposed to high sound levels, with approximately 18% reporting intention to wear them in case they were in a concert with high sound levels.

About tinnitus, the males and the students in ages 12 to 16 years report more tinnitus complaints.

The association of these factors with sex evidenced that males get exposed to noisier, hazardous hearing habits (Table 2), and females have more knowledge on the theme, as well as more positive attitudes regarding the use of hearing protection devices.

**Table 2:** Association of sex with risky noisy hearing habits (Question: ‘During the last year, I’)

**ALTERNATIVES**	***p***	**SEX**			
		**Female (n=838)**		**Male (n=997)**	
		**n**	**%**	**n**	**%**
I used headset or MP3 player	0.3722	769	91.8%	902	90.5%
I used a lawn mower	*0.0063	120	14.3%	190	19.1%
I ride a motorcycle	*0.0262	370	44.2%	493	49.4%
I went to a noisy car show	*0.0000	313	37.4%	520	52.2%
I walked in a car equipped with powerful sound	*0.0000	242	28.9%	526	52.8%
I played in a band	0.9434	85	10.1%	100	10.0%
I went to a car or motorcycle race	*0.0000	86	10.3%	247	24.8%
I went to a music show	*0.0197	329	39.3%	446	44.7%
I used fireworks	^*^0.0000	290	34.6%	504	50.6%

* Difference test of proportions at significance level of 0.05 (5%)

**Caption:** %: percentage (general responses); *p* (sex): results of '*p*' - difference between female and male

Regarding age, dividing the group of students in ages 10 to 11 years and 12 to 16 years, students in the older age range also tend to get more exposed to noisier, hazardous hearing habits (Table 3), On the other hand, younger students demonstrated more positive attitudes in relation to the use of hearing protection devices (Table 1). The analysis of general habits and knowledge on the theme did not find any statistically significant differences between the groups concerning the age ranges (Table 1).

**Table 3:** Comparison of age group with risky hearing habits (Question: ‘During the last year, I’)

**ALTERNATIVES**	***p***	**AGES (AGE)**			
		**10 to 11 (n=1058)**		**12 to 16 (n=777)**	
		**n**	**%**	**n**	**%**
I used headset or MP3 player	*0.0000	951	89.9%	270	34.7%
I used a lawn mower	0.6111	175	16.5%	135	17.4%
I ride a motorcycle	*0.0000	544	51.4%	319	41.1%
I went to a noisy car show	*0.0000	424	40.1%	409	52.6%
I walked in a car equipped with powerful sound	*0.0000	392	37.1%	376	48.4%
I played in a band	*0.0039	125	11.8%	60	7.7%
I went to a car or motorcycle race	*0.0036	168	15.9%	165	21.2%
I went to a music show	*0.0000	384	36.3%	391	50.3%
I used fireworks	0.7649	461	43.6%	333	42.9%

* Difference test of proportions at significance level of 0.05 (5%)

**Caption:** %: percentage (general responses); *p* (age): results of '*p'* - difference between the age groups lower (10 and 11 years of age) and higher (12 to 16 years of age)

## DISCUSSION

Noise has been part of individuals’ daily life and has negative impact on hearing health as well as on extra-auditory health, apart from the deleterious consequences for the general well-being and communication^(14)^. There has been increasing exposure of children and adolescents to high noise levels, reporting an increase in risk hearing habits with the noise exposure of that population^(1,15,16)^.

Concerning the risk of hearing habits to NIHL (Figure 1), the data found in the current study corroborate other studies that also used DD questionnaires. Martin et al.^(17)^ reported that about 94.5% of 1,120 American fourth graders were exposed to some kind of hazardous noise for hearing loss (ranging from 9% - those who play in a band – to 73% among those who use earphones). Study by Knobel and Lima^(13)^ conducted with a group of 220 Brazilian 4th and 5th graders, ages ranging from 8 to 11 years, observed ranges from 5.1% (play in a band), 61.9% (use of earphones) to 77.5% (attendance to noisy parties). Welch^(8)^ reported over 90% of general exposure to potentially hazardous noise sources, specially amplified music via earphones among 44 students, 14 to 17 years of age, from Auckland, New Zealand.

Some authors consider that the basic source for high incidence of hearing loss among the youth lies in the habit of listening to music using earphones at loud volume with inappropriate environmental noise levels^(18-24)^.

Study^(23)^ showed that the most important hearing-related symptoms after noise exposure were tinnitus and noise sensitivity. Regarding leisure noise exposure, listening to personal stereos/earphones was most frequently reported. The use of HPDs during most noisy activities was limited.

In addition to the attendance to concerts, use of personal stereos/ earphones, with prevalence of amplified electronic music, there are several other examples of situations and venues where the young are exposed to noise in their free, leisure time, as it was verified in this study. Additionally, other young individuals equip their cars with powerful amplifiers, reaching up to 140 decibels (dB A), while others are exposed to noise from popular Brazilian celebrations, such as the Carnival (*trio elétrico –* a popular sound truck with a band playing on top of it) and June parties. There are also the worldwide celebrations, such as the New Year’s Eve with fireworks that may cause hearing loss and tinnitus^(24)^.

Despite the evidence of hazardous noise exposure among the young, there are no regulations for the non-occupational or entertainment-noise exposure. The WHO, along with the International Telecommunication Union, recommends safe sound levels of 75 dB for 40 hours a week to children, and 80 dB for 40 hours to adults^(25)^.

Another factor considered relevant in cases of NIHL is the lack of knowledge on the theme (Table 1). Considering that this kind of hearing loss is irreversible, but its basic cause is preventable, its approach should start by the increase of the population awareness since childhood and adolescence by means of hearing health promotion and educational programs^(10)^.

In the current study, the participants admitted that they did not have knowledge on noise-related hearing loss (Table 1). Such results are worrying and raise questions on the probable reasons for this lack of knowledge on the theme. Would it be only the absence of educational campaigns at school. The adoption of specific preventive NIHL methods among children and adolescents needs to be surveyed, developed, and applied. In Brazil, a study by Knobel and Lima^(26)^ observed that 68.5% were aware that high sound levels could impair their hearing, 25% thought that children and young people were not affected by any type of hearing loss, and 20.8% of them did not believe that children could develop hearing loss. Still about this theme, when it was mentioned that children could develop hearing loss by exposure to loud sound, 5.5% of them did not believe it, and 7.4% reported that they would not do anything about that.

In the current study, only 10% of them wear or wore hearing protection when exposed to high sound levels, with about 18% reporting their intention of wearing them in case of being at a concert with high noise levels (Table 1). On the other hand, in the study^(26)^ 87.2% showed interest in learning how to protect hearing, but only 21.8% were familiar with the use of hearing protection devices. Studies that investigated the use of hearing protection by the young during noisy activities corroborated the findings that less than 10% of the adolescents wear or intend to wear them^(13,17,23,26,27)^.

Even though there have been questions addressing the lack of knowledge, some interventions have been developed to call attention to the problem and search for some changes in those behaviors and attitudes, informing about noise and its potential hazards to hearing and accessibility to hearing protection devices. Exemplifying, we have Sweden, Germany, Denmark, among other European countries. In those countries, the free supply and distribution of hearing protection devices at reduced costs are common during concerts and music festivals. Moreover, in such events, there are “hearing recovery areas”, with silent places to ‘rest your ears’, apart from several recommendations, information on the sites of the festival and at the venue, messages, signs, warnings, boxes full of hearing protection devices (with instructions on the package on how to use them), etc.^(28,29)^.

In that sense, we should advance in our country regarding the access to hearing protection (there are scarce options of proper hearing protection devices for children and adolescents), as well as the lack of knowledge or inadequate information on the use and effectiveness of such devices. That highlights even more the importance and need of implementing educational programs to raise the awareness on the theme. NIHL prevention has been recommended, but it is still far from a real inclusion in the educational planning of Brazilian schools for a number of reasons: lack of knowledge on hearing loss and the effects of high sound levels (on the part of students, teachers, parents, community in general and public authorities/ health and education managers), prioritization of other health campaigns considered more relevant and appealing (alcohol abuse, smoking, drug abuse, etc.), and the scarcity of qualified, adequately educated professionals to perform in the area^(30,31)^.

In Brazil, some health education policies^(32)^ (such as the propositions by the School Health Program/ SHP) and interventions, such as the national campaigns on the ‘International Day of Awareness on Noise/Brazil’^(33)^ objectifying the awareness of the Brazilian population on noise and its effects on health, quality of life and environment. In addition, the awareness of everyone for reducing noise from daily activities, which has been gaining visibility and supporters every year. However, according to literature, detailed, updated data unveiling the magnitude of the problem and its demand in the different regions are still missing. Studies of population profile in relation to noise are necessary and important, as such information may affect and foster greater appreciation of the theme by health and education managers, in a way that expands preventive and educational actions in the area.

Concepts of risk attitudes and behaviors are the theme of several discussions and a complex subject, as they comprise issues beyond Biology, depending on cultural, social, psychological factors and society values. Education has been demonstrated one of the effective ways to promote the change of risk behaviors, and for such, health communication theories claim that individuals must be exposed to information early in their childhood, on several occasions and by several ways^(34)^ Regarding the comparisons by sex and age ranges (Tables 1, 2 and 3), the current study found greater exposure to risk habits, with noise exposure and hearing complaints (tinnitus) among the males and older adolescents, with more positive attitudes on the use of hearing protection among younger students (Table 1), and concerning the intention of using hearing protection devices, that was verified among females and younger students as well (Table 1).

Research with an American sample and another with a Swedish sample investigated differences among the youth on their noise-related attitudes and use of hearing protection at concerts^(35)^, observing that such attitudes differed by sex and country, with American males featuring more positive attitudes in face of noise and Swedish females featuring more negative attitudes.

Contrastingly, a Brazilian study^(26)^ did not observe significant differences, regarding sex, between the same analyzed variables (risk perception/ attitudes in face of noise and strategies of hearing protection/ use of hearing protection devices).

Such differences may evolve by innumerable issues, from biological ones (higher levels of testosterone and cortisol in males, for example) to environmental and sociocultural factors^(6)^. Concerning the sociocultural scope^(36)^ study concluded that family socialization tends to stimulate a superior performance among the girls, with positive significance in their studies and at school, tougher and earlier responsibility, based on the discipline, and differing constraints between the sex as well.

The data found in that study show the dimensions of the problem beyond the lack of knowledge in the area, and open perspectives and themes for reflections to be considered on the constructions and directions of further hearing health programs to children and adolescents.

As limitations of the study, we can consider scarce depth or detailing of some topics assessed in the questionnaires, once it is a cross-sectional study with convenience sampling. Furthermore, the instrument chosen can be considered a limitation. The original English version was proposed and validated by Griest et al.^(12)^, and was translated and adapted into Brazilian Portuguese by Knobel and Lima^(13)^, but the Brazilian version has not been validated, requiring a validation study.

## CONCLUSION

The study verified that the greatest part of the children and adolescents in the assessed group evidence noise-related risk habits, reporting high percentage of hearing complaints (such as tinnitus), more evident among males, scarce knowledge on the theme or on the proper attitudes in face of the noise problem, poor intention of changing these risk behaviors, also prevalent among males and older age ranges.
